# Integrative multi-omics and network biology in cardiovascular disease: a systems-level framework for translational discovery

**DOI:** 10.3389/fsysb.2026.1897299

**Published:** 2026-09-02

**Authors:** Vanesa Brecher, Vasiliki Androutsopoulou, Serge Sicouri, Basel Ramlawi, Dimitrios V. Avgerinos, Thanos Athanasiou, Dimitrios E. Magouliotis

**Affiliations:** 1 Department of Cardiac Surgery Research, Lankenau Institute for Medical Research, Wynnewood, PA, United States; 2 Department of Cardiothoracic Surgery, Faculty of Medicine, Biopolis, University of Thessaly, Larissa, Greece; 3 Department of Cardiac Surgery, Lankenau Medical Center, Wynnewood, PA, United States; 4 Department of Cardiac Surgery, Onassis Cardiac Surgery Center, Athens, Greece; 5 Department of Surgery and Cancer, Imperial College London, London, United Kingdom

**Keywords:** cardiovascular disease, heart failure, integrative genomics, machine learning, multi-omics, network biology, systems biology, thoracic aortic aneurysm

## Abstract

Cardiovascular diseases remain a leading cause of global morbidity and mortality, driven by the complex interplay of genetic, epigenetic, transcriptomic, proteomic, and metabolic networks. Traditional reductionist approaches have inadequately captured this molecular complexity, motivating the emergence of integrative multi-omics and systems biology as foundational paradigms in cardiovascular research. This review provides a comprehensive, systems-level framework for translational discovery in cardiovascular disease, synthesizing advances in multi-omics technologies, network biology, artificial intelligence, and bioinformatics applied to conditions including thoracic aortic aneurysm, heart failure, valvular disease, and vascular remodeling. We survey the landscape of publicly available omics repositories and examine how transcriptomics, epigenomics, proteomics, and metabolomics are being integrated to decipher disease mechanisms. We outline network-based analytical frameworks encompassing protein-protein interaction networks, gene co-expression networks, hub gene analysis, and multiplex network modeling, highlighting their utility in identifying causal molecular drivers and therapeutically actionable targets. The growing contribution of machine learning, deep learning, and multimodal artificial intelligence to cardiovascular genomics is critically examined alongside challenges of interpretability and clinical validation. Finally, we address the translational interface between systems-level discovery and precision cardiovascular medicine, including polygenic risk stratification, multi-omics biomarker development, and the path from computational prediction to clinical implementation.

## Graphical Abstract

**Figure:**
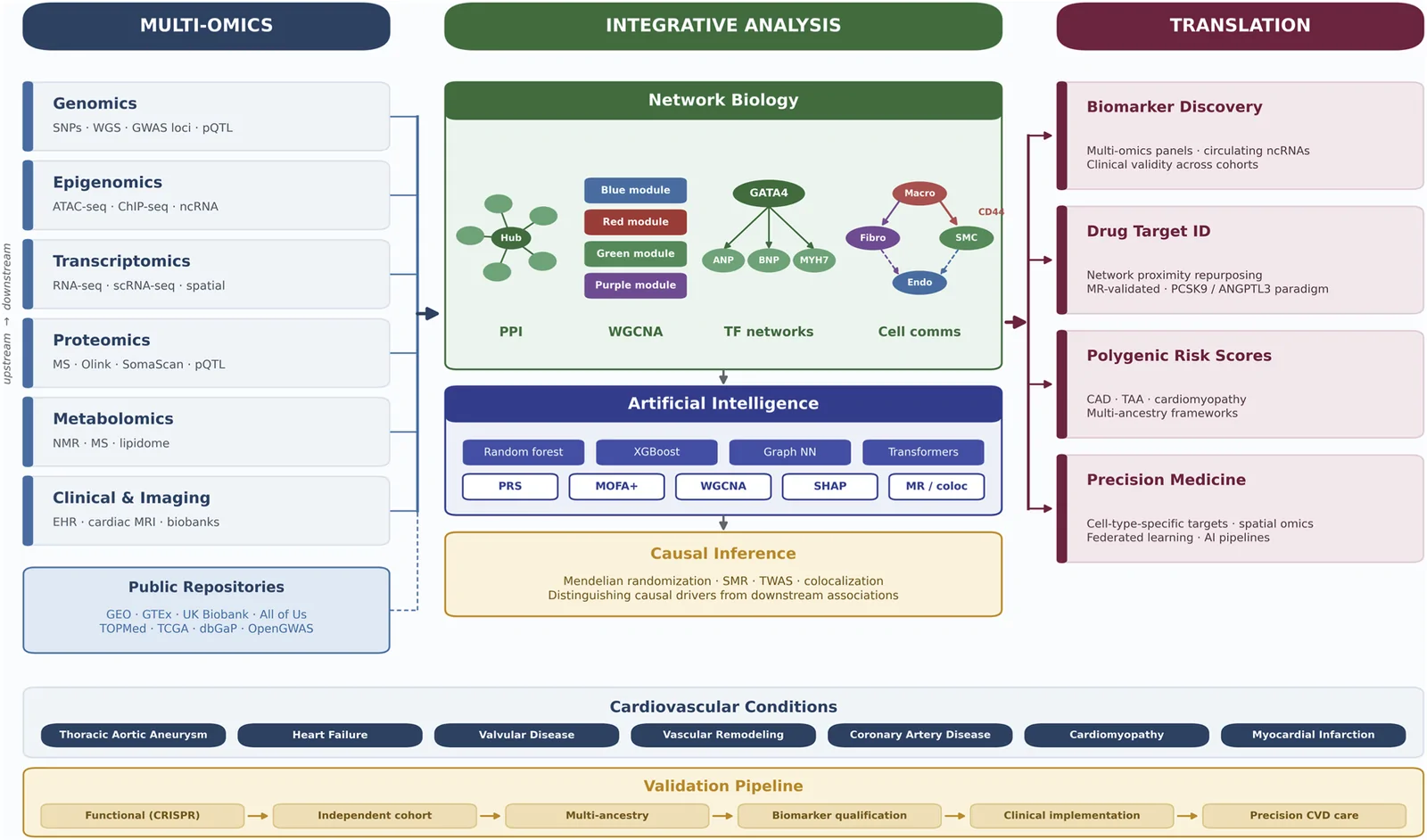
Visual summary of the systems-level framework developed in this review. Six molecular layers, ordered from upstream genomic variation through epigenomics, transcriptomics, proteomics, and metabolomics to downstream clinical and imaging phenotypes, together with major public repositories, converge on an integrative analysis stage combining network biology, artificial intelligence, and causal inference. These analyses feed four translational outputs: biomarker discovery, drug target identification and repurposing, polygenic risk stratification, and precision cardiovascular medicine, each subject to a staged validation pipeline spanning functional confirmation, independent cohort replication, multi-ancestry validation, and clinical implementation, across the cardiovascular conditions considered in this review.

## 1 Introduction

Cardiovascular diseases remain a leading cause of morbidity and mortality worldwide, with an estimated 3.7% increase in total cardiovascular disease prevalence in the United States projected by 2050 (Maddox et al., 2026). Disorders including coronary artery disease, valvular disease, aortic pathologies, and heart failure arise through complex interactions among genetic, epigenetic, metabolic, and environmental factors. Despite recent advances in cardiovascular diagnostics and therapeutics, including high-sensitivity biomarker assays, multimodal cardiac imaging, and transcatheter interventions, patients continue to experience substantial variability in disease progression, treatment response, and clinical outcomes, reflecting the molecular heterogeneity of these disorders (Greene et al., 2020). Consequently, there is growing demand for systems-level investigative strategies capable of identifying novel biomarkers, therapeutic targets, and mechanisms of disease.

Cardiovascular disease is increasingly recognized as a complex, multifactorial process driven by interactions among genetic, transcriptomic, proteomic, and metabolic networks rather than isolated gene defects. Traditional genetic studies primarily focused on identifying associations between single-nucleotide polymorphisms and disease phenotypes; however, advances in integrative genomics have shifted the field toward understanding how these variants alter gene expression, cellular signaling, and molecular network behavior within cardiovascular tissues. This transition from reductionist genetics to systems-level biology has enabled researchers to move beyond simple gene-disease associations and instead identify interacting molecular systems underlying cardiovascular pathology. Recent frameworks integrating Mendelian randomization with network biology further emphasize the importance of distinguishing causal molecular drivers from downstream biomarkers within interconnected biological systems (Yazdani et al., 2022).

The rapid expansion of high-throughput omics technologies has accelerated this transition. Technologies such as RNA sequencing, proteomics, and metabolomics now enable large-scale characterization of biological systems with increased sensitivity and resolution. Public repositories such as the Gene Expression Omnibus have expanded the accessibility of transcriptomic datasets, supporting large-scale integrative analyses across diverse cardiovascular conditions (Magouliotis et al., 2025). As multi-omics datasets continue to increase in scale and complexity, systems biology approaches have become essential for interpreting cardiovascular disease mechanisms. Computational frameworks including network analysis, machine learning, and integrative multi-omics modeling provide tools to identify biologically meaningful interactions across molecular layers (Joshi et al., 2021). Multi-omics studies integrating genomic, transcriptomic, and clinical datasets have successfully identified novel cardiovascular disease genes, validated regulatory pathways, and revealed molecular mechanisms associated with cardiac aging, vascular remodeling, and aneurysm formation (Glicksberg et al., 2019).

**FIGURE 1:**
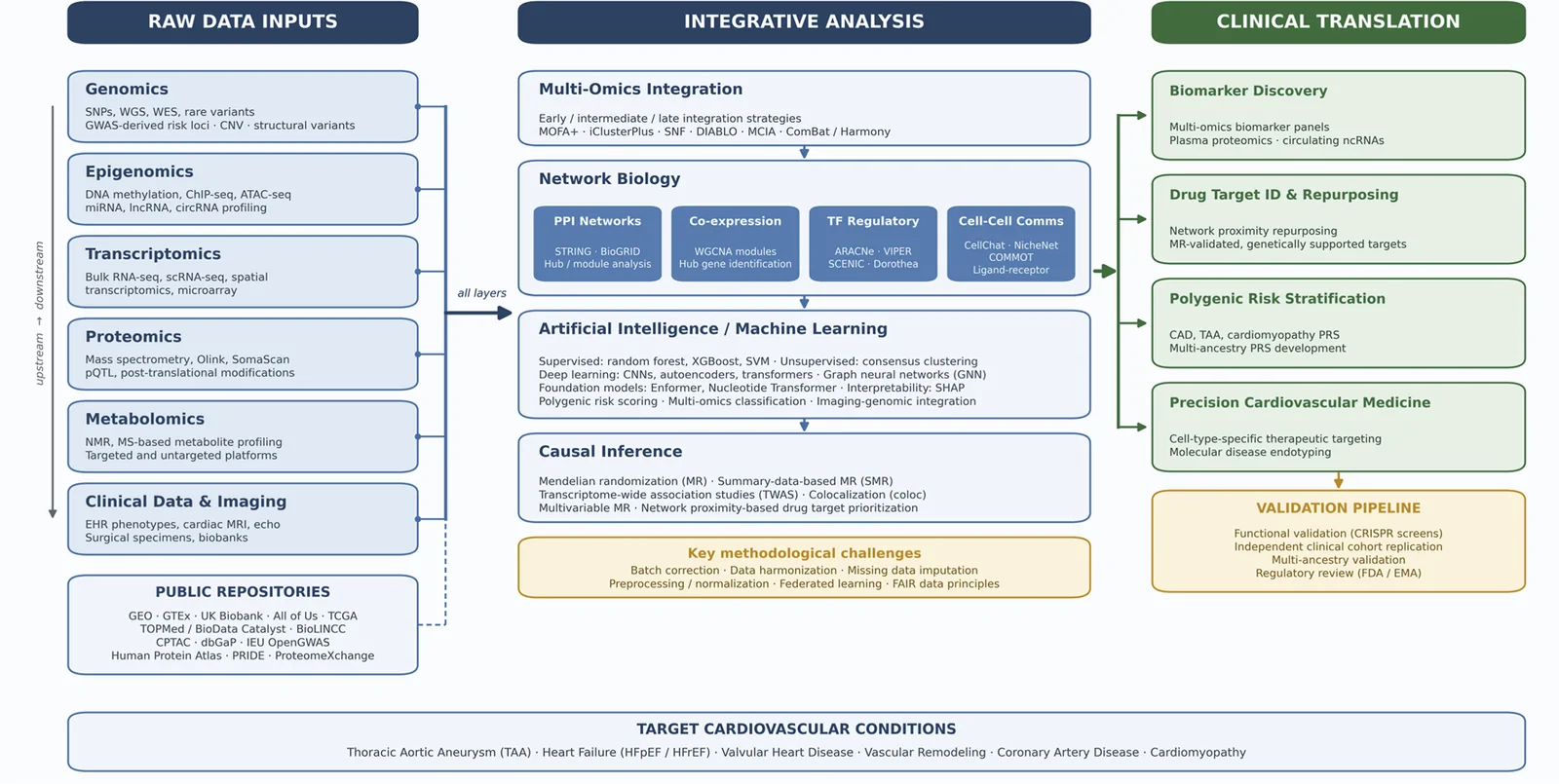
Systems-level integrative multi-omics framework for translational discovery in cardiovascular disease. Raw molecular data across six omics layers are collected from primary studies or accessed from major public repositories (GEO, GTEx, UK Biobank, All of Us, TOPMed/BioData Catalyst, TCGA, dbGaP). Omics layers are ordered to follow upstream-to-downstream biological flow, from genomic variation through epigenomics, transcriptomics, proteomics, and metabolomics to clinical and imaging phenotypes, and all six layers, together with the public repository resources, converge on the integrative analysis column rather than feeding it individually. These are integrated through complementary analytical frameworks including multi-omics factorization methods (MOFA+, SNF, iClusterPlus), network biology approaches (protein-protein interaction networks, weighted gene co-expression network analysis, transcription factor regulatory networks, cell-cell communication networks), and artificial intelligence pipelines (supervised learning, graph neural networks, deep learning, and foundation models). Causal inference methods, including Mendelian randomization, summary-data-based MR, transcriptome-wide association studies, and colocalization, are applied to distinguish causal molecular drivers from downstream associations. The output feeds a translational pipeline encompassing biomarker discovery, drug target identification, polygenic risk stratification, and ultimately precision cardiovascular medicine implementation, subject to functional validation and clinical cohort replication. AI, artificial intelligence; coloc, colocalization analysis; DL, deep learning; GEO, Gene Expression Omnibus; GNN, graph neural network; GTEx, Genotype-Tissue Expression; GWAS, genome-wide association study; LLM, large language model; MOFA+, Multi-Omics Factor Analysis version 2; MR, Mendelian randomization; PPI, protein-protein interaction; PRS, polygenic risk score; RF, random forest; SMR, summary-data-based Mendelian randomization; SNF, similarity network fusion; TCGA, The Cancer Genome Atlas; TF, transcription factor; TWAS, transcriptome-wide association study; WGCNA, weighted gene co-expression network analysis.

This review provides a systems-level framework for understanding the molecular mechanisms underlying complex cardiovascular diseases including thoracic aortic aneurysms, heart failure, valvular disease, and vascular remodeling (Figure 1). Through examination of multi-omics technologies, network biology approaches, bioinformatics pipelines, and artificial intelligence applications, this review highlights the growing utility of publicly available omics datasets for both mechanistic discovery and clinical translation.

## 2 Methods

This work is a narrative rather than a systematic review, and no formal risk-of-bias assessment or quantitative synthesis was performed. Nonetheless, to improve transparency and reproducibility, we describe our search approach. We searched PubMed/MEDLINE for English-language articles published over the last 10 years, from January 2016 through January 2026, with seminal earlier studies retained where they were foundational to a given concept, for example the original human genetic reports underpinning PCSK9 and ANGPTL3 as therapeutic targets. Search terms combined disease concepts (thoracic aortic aneurysm, aortic dissection, heart failure, valvular heart disease, coronary artery disease, cardiomyopathy, vascular remodeling, cardiovascular disease) with methodological concepts (multi-omics, integrative genomics, genome-wide association study, transcriptomics, single-cell RNA sequencing, spatial transcriptomics, epigenomics, DNA methylation, non-coding RNA, proteomics, pQTL, metabolomics, network biology, protein-protein interaction, WGCNA, machine learning, deep learning, Mendelian randomization, polygenic risk score) using Boolean operators and relevant MeSH terms. Reference lists of included articles and relevant reviews were hand-searched to identify additional sources not captured by the primary search. Articles were eligible if they reported original omics, network-based, or artificial intelligence analyses in human cardiovascular disease, described methodological frameworks for multi-omics integration, or described major public repositories or biobank resources. We excluded studies without a cardiovascular focus, those reporting exclusively animal or *in vitro* data without human translational relevance, conference abstracts, and articles not available in English. Given the breadth of the field, studies were prioritized for inclusion on the basis of methodological rigor, sample size, novelty of the analytical approach, translational relevance to clinical cardiovascular practice, and citation impact, with preference given to landmark studies, large-scale consortium analyses, and reports published within the last 5 years where the underlying technology or evidence base has evolved rapidly.

## 3 The landscape of public omics resources

### 3.1 Major data repositories and their cardiovascular relevance

The development of large-scale public omics repositories has transformed biomedical and cardiovascular research by providing widespread access to high-throughput molecular datasets (Table 1). The Gene Expression Omnibus (GEO), developed by the National Center for Biotechnology Information, stores RNA sequencing, microarray, and epigenomic datasets generated across diverse experimental conditions and disease states (Barrett et al., 2013). The Cancer Genome Atlas (TCGA) provides comprehensive genomic, transcriptomic, epigenetic, and clinical datasets enabling large-scale characterization of molecular alterations with relevance to cardiovascular biology (National Cancer Institute, 2026). The Genotype-Tissue Expression (GTEx) project links genetic variation to tissue-specific gene expression patterns across numerous human tissues, offering a reference atlas for cardiovascular eQTL analyses (GTEx Consortium, 2026). The UK Biobank, with its 500,000 participant cohort including cardiac MRI, WES, and plasma proteomics sub-studies, has emerged as the most powerful platform for large-scale cardiovascular genomics (Sudlow et al., 2015). Beyond these foundational resources, the Clinical Proteomic Tumor Analysis Consortium (CPTAC), Human Protein Atlas, PhosphoSitePlus, PRIDE, dbGaP, and the IEU OpenGWAS platform further extend opportunities for proteogenomic, epigenomic, and causal inference investigations into cardiovascular pathology. The All of Us Research Program, which has generated clinical-grade whole-genome sequences for over 245,000 participants with linked electronic health records, is distinguished by the deliberate enrollment of communities historically under-represented in genomic research, and is expected to exceed the UK Biobank in genomic scale as enrollment continues toward its one-million-participant target (All of Us Research Program Genomics Investigators, 2024). Complementary NHLBI-supported infrastructure, including the Trans-Omics for Precision Medicine (TOPMed) program and its BioData Catalyst analysis ecosystem, the Biologic Specimen and Data Repository Information Coordinating Center (BioLINCC), and major NHLBI-funded cardiovascular cohorts such as the Framingham Heart Study and the Multi-Ethnic Study of Atherosclerosis (MESA), extends this landscape by linking deep multi-omics characterization to longitudinal clinical phenotypes, imaging, and biospecimens across ancestrally diverse populations.

**TABLE 1:** Major public omics repositories: primary data types, cardiovascular disease applications, key features, and access information.

Repository	Primary data types	CVD Applications	Key features	Access
Gene expression omnibus (GEO)	RNA-seq, microarray, ChIP-seq, ATAC-seq, methylation arrays	TAA, heart failure, valve disease, vascular remodeling, cardiomyopathy	Largest transcriptomic archive; supports GEO2R for differential expression; widely cited in secondary analyses	https://www.ncbi.nlm.nih.gov/geo/
The cancer genome atlas (TCGA)	WGS, WES, RNA-seq, methylation, CNV, miRNA, proteomics	Cancer-cardiovascular intersection; vascular tumor biology; drug cardiotoxicity genomics	Multi-omics per tumor type; paired normal tissue data; integrated clinical annotations	https://www.cancer.gov/tcga/
GTEx	RNA-seq across 54 tissues; genotype data; sQTL, eQTL	Cardiac and aortic eQTL mapping; tissue-specific expression of CVD risk genes	Reference for tissue-specific gene regulation; ∼1,000 donors; includes heart, aorta, coronary artery	https://www.gtexportal.org/
UK Biobank	WES/WGS, genotyping, metabolomics, proteomics, imaging, EHR	CAD PRS; aortic dimensions; cardiomyopathy genetics; heart failure risk factors	500,000 participants; longitudinal follow-up; cardiac MRI sub-study; linked hospital records	https://www.ukbiobank.ac.uk/
All of us research program	WGS, genotyping, EHR, survey, wearable and physical measurement data	Multi-ancestry PRS development and validation; equitable cardiovascular risk prediction	245,000+ diverse whole genomes released (target 1,000,000 participants); 77% from communities historically under-represented in genomics; linked longitudinal EHR	https://www.researchallofus.org/
CPTAC	Mass spectrometry proteomics, phosphoproteomics, glycoproteomics	Cancer-cardiac intersection; chemotherapy cardiotoxicity proteome	Deep proteogenomic integration; paired RNA-seq and proteomics per sample	https://www.proteomics.cancer.gov/
Human protein atlas	Protein IHC, scRNA-seq, spatial transcriptomics, plasma proteomics	Cardiac cell-type protein expression; aortic tissue profiling; circulating protein biomarkers	Tissue and cell type resolution; integrated RNA and protein evidence; freely browsable	https://www.proteinatlas.org/
PhosphoSitePlus	Post-translational modifications (phosphorylation, ubiquitination, acetylation)	Cardiac signaling network analysis; kinase substrate mapping in hypertrophy and failure	Manually curated PTM database; supports network analysis of signaling cascades	https://www.phosphosite.org/
PRIDE/ProteomeXchange	Mass spectrometry raw data and processed proteomics datasets	Cardiovascular proteomics repository; circulating biomarker datasets	Primary deposition archive for proteomics; supports FAIR data principles	https://www.ebi.ac.uk/pride/
dbGaP	GWAS summary statistics, WGS, WES, phenotype data	Cardiac GWAS; aortic disease genetics; cardiomyopathy variant discovery	NIH-managed controlled-access repository; large population cohorts	https://www.ncbi.nlm.nih.gov/gap/
IEU OpenGWAS	GWAS summary statistics across thousands of traits	MR for CVD risk factor causal inference; multivariable MR	MR-base platform integration; supports two-sample MR pipelines; continuously updated	https://www.gwas.mrcieu.ac.uk/

Abbreviations: ATAC-seq, assay for transposase-accessible chromatin sequencing; CAD, coronary artery disease; ChIP-seq, chromatin immunoprecipitation sequencing; CNV, copy number variation; CVD, cardiovascular disease; eQTL, expression quantitative trait locus; GEO, gene expression omnibus; GTEx, Genotype-Tissue Expression; GWAS, genome-wide association study; IHC, immunohistochemistry; MR, mendelian randomization; PTM, post-translational modification; PRS, polygenic risk score; scRNA-seq, single-cell RNA, sequencing; SMR, summary-data-based Mendelian randomization; sQTL, splicing quantitative trait locus; TCGA, the cancer genome atlas; WES, whole exome sequencing; WGS, whole genome sequencing.

### 3.2 Multi-omics analysis platforms: turning open-access datasets into discovery and reproducibility

While Section 3.1 catalogs the repositories that store cardiovascular omics data, realizing their translational value depends equally on the computational platforms used to analyze and integrate them. Open-access omics datasets have enabled increasingly sophisticated systems-level analyses by supporting integration of multiple molecular layers across large patient populations. Advances in single-cell multi-omics technologies now allow simultaneous characterization of genomic, transcriptomic, epigenomic, and proteomic information within individual cells, providing high-resolution insight into cellular heterogeneity and disease mechanisms (Baysoy et al., 2023). In cardiovascular disease research, integrative multi-omics approaches have facilitated the identification of disease-associated regulatory pathways and the discovery of novel biomarkers through analysis of molecular activity within clinically relevant tissues.

Tools such as OmicsNet and NetworkAnalyst streamline workflows involving multiple omics layers by providing accessible platforms for network modeling, visualization, and statistical analysis (Zhou et al., 2022). The increasing availability of such computational platforms, combined with expanded repository infrastructure, has accelerated reproducibility, large-scale discovery, and collaborative research, while also highlighting ongoing challenges related to data harmonization, batch effect correction, and interpretation of multifactorial cardiovascular disease mechanisms (Luo et al., 2017).

### 3.3 Genomic discoveries in cardiovascular disease: a GWAS foundation

Genome-wide association studies (GWAS) provide the foundational layer upon which the multi-omics frameworks described throughout this review are built, anchoring transcriptomic, epigenomic, proteomic, and network-based findings to inherited genetic risk. Large-scale GWAS meta-analyses combining the UK Biobank with the CARDIoGRAMplusC4D consortium, spanning over 800,000 individuals, have identified genetic determinants of myocardial infarction and coronary artery disease and helped distinguish atherosclerosis-susceptibility loci from loci that specifically predispose to plaque rupture and thrombosis (Hartiala et al., 2021). In thoracic aortic aneurysm and dissection, a GWAS conducted through the Million Veteran Program identified 21 genome-wide significant risk loci, 17 of which were previously unreported, and provided human genetic evidence that thoracic aortic disease is mechanistically distinct from atherosclerotic vascular disease rather than a variant of the same process (Klarin et al., 2023). These discovery-stage findings motivate the downstream analyses detailed throughout this review: candidate loci nominated by GWAS are prioritized for eQTL mapping and network placement (Sections 4 and 7), evaluated for regulatory mechanism through epigenomic profiling (Section 5), tested for causal effect through Mendelian randomization (Section 9.2), and ultimately incorporated into polygenic risk scores for clinical risk stratification (Section 10.3). The scale of contemporary cardiovascular GWAS, increasingly enabled by biobanks such as the UK Biobank and the All of Us Research Program (Section 3.1), continues to expand the catalog of genomic loci available for this discovery-to-translation pipeline.

## 4 Transcriptomics in cardiovascular disease

### 4.1 RNA sequencing and microarray technologies

Transcriptomic technologies have fundamentally transformed cardiovascular disease research by enabling large-scale characterization of gene expression patterns across diverse cardiac and vascular cell populations. Early transcriptomic studies relied on microarray platforms, generating extensive legacy datasets that remain valuable for comparative analyses across cardiovascular conditions. More recently, RNA sequencing (RNA-seq) and single-cell RNA sequencing (scRNA-seq) have shifted the focus from population-level expression profiling to high-resolution mapping of cellular heterogeneity and dynamic transcriptional states (Paik et al., 2020). Unlike microarrays, RNA-seq does not depend on predefined probes and allows detection of low-abundance transcripts, alternative splice variants, and previously uncharacterized genes. scRNA-seq further enables characterization of cellular heterogeneity within cardiovascular tissues by distinguishing transcriptionally distinct populations of cardiomyocytes, fibroblasts, endothelial cells, smooth muscle cells, and immune cells that cannot be resolved using bulk analysis (Paik et al., 2020).

Advances in computational analysis methods, including clustering algorithms, trajectory inference, t-SNE, and UMAP, have improved the interpretation of high-dimensional transcriptomic datasets. More recently, spatial transcriptomics combined with multi-omics approaches has enabled the spatial mapping of gene expression within cardiovascular tissues, revealing how cell-cell communication networks and tissue architecture jointly contribute to disease, and supporting the development of precision cardiovascular medicine (Paik et al., 2020).

### 4.2 Applications across major cardiovascular conditions

#### 4.2.1 Heart failure

Transcriptomic analyses have provided important insights into the molecular mechanisms underlying heart failure and cardiac remodeling. Recent studies in heart failure with preserved ejection fraction (HFpEF) identified metabolically driven fibroblast activation and extracellular matrix remodeling as central pathogenic processes, with angiopoietin-like 4 (ANGPTL4) emerging as a key regulator linking lipid metabolism, inflammation, and fibrosis (Lanzer et al., 2024). Increased collagen IV expression further suggests that HFpEF-associated fibrosis involves basement membrane remodeling in addition to traditional interstitial collagen deposition. RNA-seq studies have also demonstrated that relaxin therapy reverses maladaptive inflammatory and fibrotic transcriptional programs associated with aging-related heart failure and atrial fibrillation, while revealing important sex-specific differences in cytokine signaling and gene expression regulation (Martin et al., 2018).

#### 4.2.2 Valvular disease

In valve disease, scRNA-seq studies identified distinct valvular interstitial cell (VIC) phenotypes, including quiescent, activated, and osteogenic VIC populations, associated with fibrosis and calcification (Qin and Bäck, 2026). These analyses also revealed significant immune cell infiltration and extensive signaling crosstalk among valvular endothelial cells, stromal cells, and inflammatory populations, supporting the concept that valvular degeneration involves coordinated immune-mediated remodeling rather than passive tissue degeneration alone.

#### 4.2.3 Vascular remodeling and myocardial infarction

In vascular remodeling following myocardial infarction, integration of scRNA-seq with spatial transcriptomics demonstrated that remodeling is driven by coordinated gene expression networks spanning endothelial cells, fibroblasts, pericytes, and immune cells within anatomically distinct regions such as the infarct core and border zone (Kuppe et al., 2022). These findings highlight the importance of spatially constrained cell-cell communication networks in regulating angiogenesis, fibrosis, and inflammatory responses during tissue repair.

#### 4.2.4 Thoracic aortic aneurysm

Transcriptomics has substantially improved understanding of thoracic aortic aneurysm (TAA) pathogenesis by revealing interconnected inflammatory, extracellular matrix, and smooth muscle cell signaling networks. Recent studies demonstrated that aneurysm formation is associated with the collapse of smooth muscle cell contractile gene programs alongside the activation of inflammatory and proteolytic pathways (Zhao et al., 2025). Distinct macrophage subpopulations characterized by high GPNMB expression were identified as key network drivers that interact directly with smooth muscle cells through CD44-mediated signaling, promoting phenotypic switching, cytokine production, and extracellular matrix degradation. Network-based analyses further demonstrate that TAA progression is driven by coordinated dysregulation of endothelial function, smooth muscle remodeling, and extracellular matrix homeostasis rather than isolated gene abnormalities alone (Ju et al., 2026). Studies from our group have identified the significant dysregulation of gap junction proteins and aquaporin channels as functionally important transcriptomic signatures in TAA, uncovering novel molecular targets for future investigation (Magouliotis et al., 2026a; Magouliotis et al., 2026b).

Across each of these conditions, transcriptomic signatures describe which genes are dysregulated but not why; the epigenomic mechanisms that establish and maintain these transcriptional states are considered next.

## 5 Epigenomics in cardiovascular disease

### 5.1 DNA methylation and cardiovascular phenotypes

DNA methylation represents one of the best-characterized epigenetic mechanisms regulating cardiovascular gene expression. Epigenome-wide association studies (EWAS) have identified differentially methylated regions (DMRs) associated with coronary artery disease, heart failure, and aortic pathology, often at loci not captured by GWAS (Nakatochi et al., 2017). Methylation patterns at key regulatory elements govern the transcriptional activity of genes involved in vascular smooth muscle cell differentiation, endothelial function, and cardiomyocyte identity. The concept of an epigenetic aging clock, most prominently the Horvath and Hannum clocks derived from CpG methylation arrays, has been applied to cardiovascular risk stratification, with accelerated epigenetic aging independently predicting incident cardiovascular events beyond traditional risk factors (Horvath and Raj, 2018). Tissue-specific methylation patterns in aortic wall, myocardium, and circulating immune cells have been linked to distinct phases of cardiovascular disease progression, and integration with transcriptomic data has helped prioritize functionally relevant DMRs over statistical associations alone (Liu et al., 2024).

### 5.2 Histone modification and chromatin accessibility

Chromatin-level regulation through histone modification and chromatin accessibility constitutes a second major epigenomic layer relevant to cardiovascular disease. ChIP-seq profiling of active enhancer marks (H3K27ac, H3K4me1) and promoter marks (H3K4me3) in cardiac and vascular tissues has revealed extensive enhancer remodeling during pathological hypertrophy, fibrosis, and ischemic injury (Papait et al., 2013). ATAC-seq, which maps open chromatin regions, has been applied in cardiomyocytes, cardiac fibroblasts, and aortic smooth muscle cells to characterize the regulatory landscape underlying disease-associated gene programs. Integration of ATAC-seq with transcription factor binding motif analysis enables inference of the master regulators driving chromatin remodeling in cardiovascular disease, complementing TF network approaches derived from transcriptomic data (Corces et al., 2018). Single-cell ATAC-seq has further enabled chromatin accessibility mapping at cellular resolution within complex cardiovascular tissues, revealing cell-type-specific regulatory programs that are invisible in bulk assays.

### 5.3 Non-coding RNAs: microRNAs, long non-coding RNAs, and circular RNAs

Non-coding RNAs (ncRNAs) constitute a functionally diverse epigenomic regulatory layer with broad relevance to cardiovascular pathology. MicroRNAs (miRNAs) post-transcriptionally regulate gene expression by binding to the 3′-UTR of target mRNAs, and specific miRNA signatures have been characterized in myocardial infarction, heart failure, aortic disease, and vascular remodeling. The miR-29 family, which targets multiple extracellular matrix genes, is consistently downregulated in fibrotic cardiac and aortic tissue, and its restoration has been proposed as a therapeutic strategy (Tao et al., 2015). Long non-coding RNAs (lncRNAs) including MIAT, MALAT1, and NEAT1 have been implicated in cardiac hypertrophy, atherosclerosis, and ischemic injury through regulation of chromatin architecture, transcription factor activity, and competing endogenous RNA networks (Piccoli et al., 2015). Circular RNAs (circRNAs), formed by back-splicing events, have emerged as stable, tissue-specific regulators of cardiovascular gene expression with potential as diagnostic biomarkers. Integration of ncRNA expression data with protein-coding transcriptomic and network analyses adds a further regulatory dimension to systems-level cardiovascular models (Holdt et al., 2016).

Transcriptomic and epigenomic layers together describe gene regulation, but the functional consequences of cardiovascular disease are ultimately executed by proteins and metabolites, the layers addressed next.

## 6 Proteomics and metabolomics integration

### 6.1 Cardiovascular proteomics: platforms and applications

Proteomics bridges the gap between transcriptomic findings and functional biological activity, as protein abundance, modification, and localization ultimately determine cellular phenotype. Two broad platform families dominate cardiovascular proteomics: mass spectrometry (MS)-based approaches and affinity-based multiplexed protein assays. Liquid chromatography-tandem MS enables deep proteome coverage with quantification of thousands of proteins per sample, and has been applied to characterize the cardiac, aortic, and plasma proteomes in heart failure, aortic aneurysm, and atherosclerosis (Doll et al., 2017). Affinity-based platforms, including the Olink proximity extension assay and the SomaScan aptamer-based assay, enable high-throughput quantification of hundreds to thousands of proteins in plasma, making them particularly suited to large population cohort studies such as the UK Biobank Pharma Proteomics Project (Sun et al., 2023). The latter has already mapped proteomic signatures for dozens of cardiovascular conditions, providing an unprecedented catalog of circulating protein biomarkers with potential clinical utility.

Post-translational modifications (PTMs) represent an additional proteomics dimension of cardiovascular relevance. Phosphoproteomics has revealed kinase-substrate networks underlying cardiac hypertrophy signaling, while ubiquitinomics has characterized protein degradation dynamics in ischemic injury and heart failure. The PhosphoSitePlus database consolidates post-translational modification evidence from the literature to support network-level analysis of signaling cascades in cardiovascular pathology.

### 6.2 Metabolomics in cardiovascular stratification

Metabolomics captures the downstream biochemical output of gene expression, protein activity, and environmental exposure, providing a functional readout of cardiovascular system state. Untargeted metabolomics using nuclear magnetic resonance spectroscopy or MS platforms profiles hundreds to thousands of metabolites simultaneously, while targeted panels provide precise quantification of specific metabolite classes. Fatty acid oxidation impairment and ketone body metabolism have been consistently implicated in the metabolomic signature of heart failure, and integration of metabolomics with transcriptomic data has helped dissect the mechanistic basis of metabolic flexibility loss in failing myocardium (Lopaschuk et al., 2021). Plasma amino acid profiles, including elevated branched-chain amino acids, have been identified as independent predictors of incident cardiovascular events in large population cohorts (Ur Rehman et al., 2025). In aortic disease, metabolomic profiling of aortic wall tissue has identified lipid peroxidation and oxidative stress signatures associated with aneurysm expansion, complementing transcriptomic evidence of inflammatory network activation.

### 6.3 Proteogenomics: bridging genome and proteome

Proteogenomics, the integration of genomic and proteomic data, provides a powerful framework for identifying causal protein mediators of genetic cardiovascular risk. Protein quantitative trait locus (pQTL) studies map genetic variants that influence protein abundance, enabling Mendelian randomization analyses that test causal effects of circulating proteins on cardiovascular outcomes (Sun et al., 2018). Large-scale pQTL resources from deCODE Genetics, the INTERVAL study, and the UK Biobank Pharma Proteomics Project have identified thousands of pQTLs, several of which colocalize with GWAS signals for coronary artery disease, heart failure, and aortic valve disease, providing direct evidence for genetically mediated protein-disease relationships. This proteogenomic framework is increasingly recognized as a high-yield strategy for therapeutic target prioritization, as proteins with genetic support for cardiovascular causality are substantially more likely to succeed in clinical development (Nelson et al., 2015).

Genomic, transcriptomic, epigenomic, proteomic, and metabolomic findings each identify disease-relevant molecules in isolation; network biology, discussed next, provides the formal framework for situating these molecules within the interacting systems through which cardiovascular disease actually operates.

## 7 Network biology frameworks in cardiovascular research

### 7.1 Protein-protein interaction networks

Protein-protein interaction (PPI) networks map the physical and functional associations among proteins, providing a topological representation of the cardiovascular molecular interactome. Major PPI databases including STRING, BioGRID, and IntAct compile interaction evidence from experimental assays, literature curation, and computational prediction, enabling construction of tissue-specific cardiovascular interactomes (Szklarczyk et al., 2021). Within these networks, topological analysis identifies hub proteins (high degree), bottleneck proteins (high betweenness centrality), and functionally dense modules, each with distinct biological and therapeutic significance. Hub proteins in the cardiac interactome include key signaling mediators such as TP53, AKT1, and MAPK1, whose dysregulation contributes to cardiomyocyte apoptosis, hypertrophic signaling, and stress response in heart failure (Bossi and Lehner, 2009). In thoracic aortic aneurysm, PPI network analysis of differentially expressed genes has identified extracellular matrix remodeling hubs, including MMP2, FBN1, and COL3A1, as central nodes linking smooth muscle cell phenotypic switching to structural aortic wall degradation, with direct implications for diagnostic biomarker discovery (Magouliotis et al., 2026b).

### 7.2 Gene co-expression networks and WGCNA

Weighted gene co-expression network analysis (WGCNA) constructs networks of highly correlated genes from transcriptomic data, grouping them into modules that represent co-regulated biological programs (Langfelder and Horvath, 2008). Module eigengenes, representative expression summaries per module, are then correlated with clinical traits or disease phenotypes to identify modules of functional relevance. This approach has been widely applied in cardiovascular disease: in heart failure, WGCNA has identified fibrosis-associated modules enriched for TGF-beta signaling and extracellular matrix genes, while in thoracic aortic aneurysm, modules reflecting smooth muscle cell contractile gene collapse have been consistently identified across independent GEO datasets (Zhang et al., 2023). Hub genes within disease-relevant modules, identified by high module membership and gene significance scores, represent high-priority candidates for functional validation and therapeutic targeting. The scalability of WGCNA to large transcriptomic datasets, combined with its compatibility with public GEO repositories, makes it a particularly accessible entry point for cardiovascular systems biology.

### 7.3 Transcription factor and regulatory networks

Transcription factor (TF) regulatory networks reconstruct the upstream regulatory logic governing cardiovascular gene expression programs. Methods including ARACNe (Algorithm for the Reconstruction of Accurate Cellular Networks), VIPER (Virtual Inference of Protein-activity by Enriched Regulon analysis), Dorothea, and SCENIC infer TF activity from the expression patterns of their downstream target genes (regulons), enabling identification of master regulators driving disease-associated transcriptional states (Alvarez et al., 2016). In cardiac remodeling, TF network analyses have consistently implicated GATA4, MEF2C, NFAT, and NF-kB as key regulatory hubs, while in vascular disease, KLF4 and SP1 emerge as central mediators of smooth muscle cell phenotypic transitions (Haldar et al., 2007). The integration of TF network inference with chromatin accessibility data from ATAC-seq further refines the identification of active regulatory elements and their controlling TFs within cardiovascular tissues, providing mechanistic depth beyond differential expression analyses alone.

### 7.4 Multiplex and cell-cell communication networks

Single-cell transcriptomic data has enabled a new class of network analysis focused on intercellular communication. Tools including CellChat, NicheNet, and COMMOT infer ligand-receptor interaction networks from scRNA-seq data, mapping the directionality and strength of signaling between distinct cell populations within cardiovascular tissues (Jin et al., 2021). In thoracic aortic aneurysm, cell-cell communication network analyses have revealed extensive crosstalk between GPNMB-high macrophages and smooth muscle cells, mediated by CD44-dependent signaling, as a central mechanism driving smooth muscle cell phenotypic switching and extracellular matrix degradation (Zhao et al., 2025). In post-infarction remodeling, spatial transcriptomics combined with cell-cell communication network analysis has identified region-specific signaling programs that coordinate angiogenesis and fibrosis within the border zone (Kuppe et al., 2022). Multiplex network frameworks that integrate PPI, co-expression, and cell-cell communication networks into unified multilayer representations provide the most comprehensive systems-level view of cardiovascular disease mechanisms currently achievable.

### 7.5 Network topology and drug target identification

Network topology analysis offers a principled framework for identifying therapeutically actionable targets within cardiovascular disease networks. The network proximity principle posits that disease genes and drug targets tend to cluster within the same network neighborhood, enabling computational drug repurposing by measuring the topological proximity between approved drug targets and cardiovascular disease modules (Guney et al., 2016). This approach has identified several candidate repurposing opportunities for heart failure and coronary artery disease, including drugs originally developed for oncology or metabolic disease. In one illustrative application, network proximity analysis of the protein-protein interactome screened over 900 FDA-approved drugs against coronary artery disease modules and generated candidate associations that were subsequently tested in longitudinal healthcare data from over 220 million patients; this pipeline validated hydroxychloroquine as associated with reduced coronary artery disease risk, a finding further supported by *in vitro* evidence that it attenuates cytokine-mediated activation of human aortic endothelial cells, illustrating how network-nominated candidates can be carried through population-level and mechanistic validation (Cheng et al., 2018). Essential hub genes, those whose removal disrupts large fractions of the network, warrant caution as therapeutic targets due to potential off-target toxicity, while peripheral or module-specific hubs may offer more selective intervention points. Integration of network proximity analysis with MR-validated causal targets provides the highest-confidence prioritization framework for cardiovascular drug discovery.

### 7.6 Limitations and open challenges in network biology frameworks

Despite their utility, network biology frameworks carry limitations that temper their translational reliability. Public interaction databases such as STRING and BioGRID are incomplete and unevenly annotated, with well-studied genes overrepresented and tissue-specific interactions systematically underrepresented, so that network topology can reflect research attention as much as biology. Most PPI and co-expression networks are static representations built from steady-state or cross-sectional data, whereas cardiovascular disease processes such as aneurysm progression and post-infarction remodeling are inherently dynamic; capturing this requires temporally resolved network reconstruction, an area still in early development. Network structure is also highly sensitive to the reference interactome, edge-confidence thresholds, and clustering algorithm chosen, so that hub and module assignments generated from the same expression data can vary substantially across pipelines, limiting reproducibility across studies. Scalability is a further constraint: multiplex networks integrating PPI, co-expression, and cell-cell communication layers become computationally demanding as single-cell cohorts grow, and the multi-institutional data pooling often required to achieve adequate power raises patient privacy and data-governance barriers. Federated learning, in which models are trained across institutional datasets without centralizing patient-level data, offers a pathway to build larger and more representative cardiovascular networks while keeping data within its institution of origin; this approach is discussed further, alongside other integration strategies, in Section 9.3. Finally, network-nominated hubs and drug targets require independent experimental or clinical validation, since topological centrality alone does not establish causality or therapeutic tractability.

**FIGURE 2:**
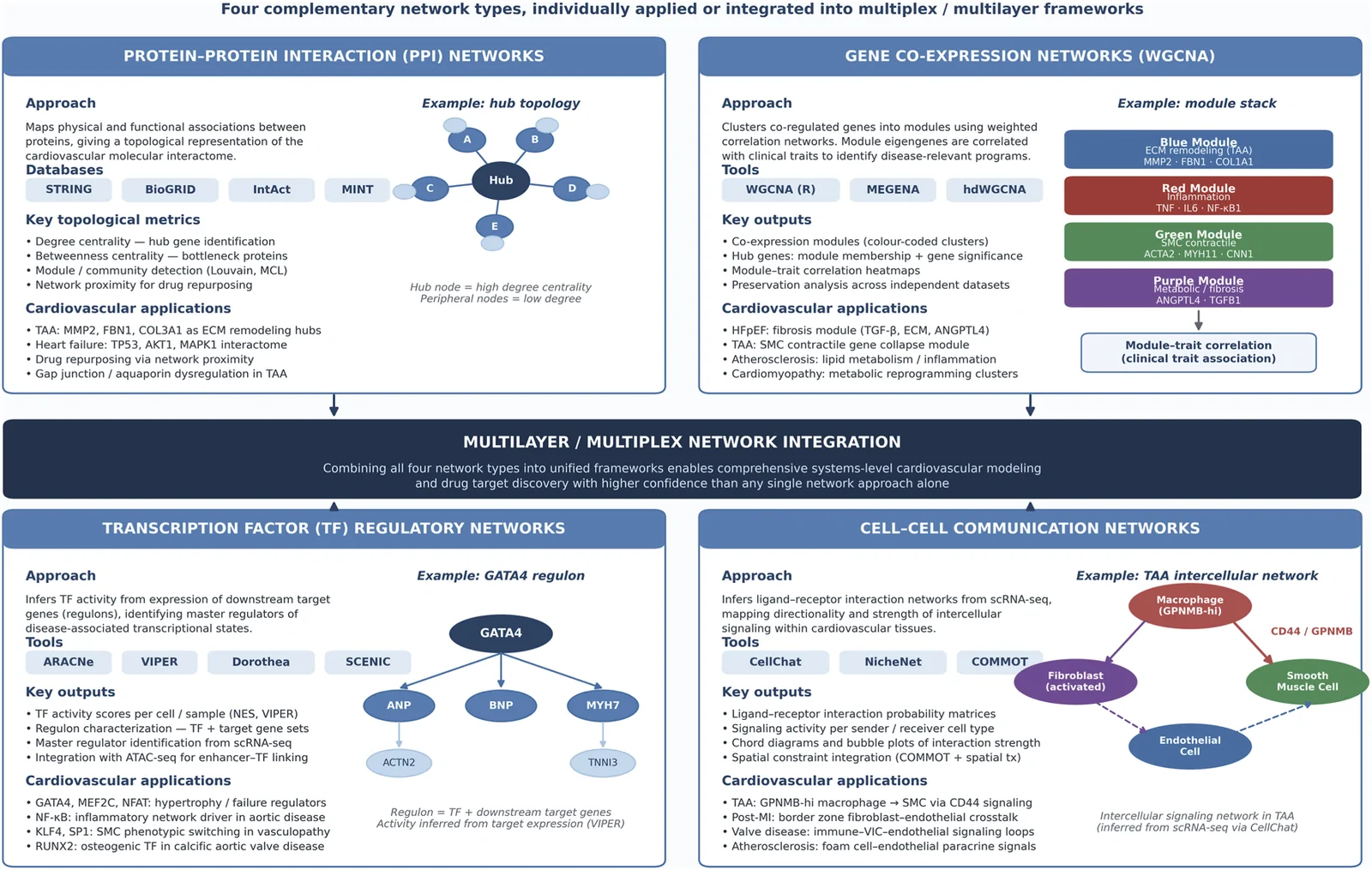
Network biology analytical frameworks applied in cardiovascular disease research. Four complementary network types are illustrated. (Upper left) Protein-protein interaction (PPI) networks, constructed from curated databases including STRING, BioGRID, and IntAct, enable topological analysis of hub genes, bottleneck proteins, and functional modules, supporting drug target prioritization and disease interactome characterization. (Upper right) Gene co-expression networks, most commonly derived through weighted gene co-expression network analysis (WGCNA), identify clusters of co-regulated genes (modules) that correlate with disease phenotypes or clinical traits, facilitating discovery of disease-relevant gene programs such as fibrosis modules in HFpEF or smooth muscle cell contractile gene collapse in thoracic aortic aneurysm; the module-trait correlation step, by which module eigengenes are related to clinical phenotypes, is shown beneath the module stack. (Lower left) Transcription factor regulatory networks, inferred by tools including VIPER, ARACNe, Dorothea, and SCENIC, estimate the transcriptional activity of master regulators from the expression patterns of their downstream targets, enabling identification of dysregulated transcription factors driving cardiovascular remodeling. (Lower right) Cell-cell communication networks, inferred from single-cell RNA sequencing using platforms including CellChat, NicheNet, and COMMOT, map intercellular ligand-receptor interactions and their downstream signaling consequences within cardiovascular tissues. Collectively, these four network types can be integrated into multiplex or multilayer network frameworks, providing a comprehensive systems-level view of cardiovascular disease mechanisms and therapeutically actionable nodes. ARACNe, algorithm for the reconstruction of accurate cellular networks; HFpEF, heart failure with preserved ejection fraction; MI, myocardial infarction; PPI, protein-protein interaction; scRNA-seq, single-cell RNA sequencing; SCENIC, single-cell regulatory network inference and clustering; SMC, smooth muscle cell; TAA, thoracic aortic aneurysm; TF, transcription factor; VIPER, virtual inference of protein-activity by enriched regulon analysis; WGCNA, weighted gene co-expression network analysis.

For a schematic representation of the four main network biology frameworks and their cardiovascular applications, see Figure 2.

## 8 Artificial intelligence and machine learning in cardiovascular genomics

### 8.1 Supervised learning for disease classification and risk prediction

Supervised machine learning methods have been extensively applied to cardiovascular omics data for disease classification, phenotype prediction, and risk stratification (Table 2). Random forest and gradient boosting algorithms, including XGBoost and LightGBM, handle high-dimensional omics features with robustness to overfitting and provide built-in feature importance metrics that facilitate biological interpretation (Christodoulou et al., 2019). These methods have been applied to multi-omics cardiovascular datasets for tasks including heart failure subtype classification, myocardial infarction diagnosis from transcriptomic signatures, and post-operative outcome prediction in aortic surgery. Polygenic risk scoring represents a clinically mature application of regularized regression (LASSO, ridge, elastic net) to genome-wide variant data, and PRS models for coronary artery disease, aortic aneurysm, and cardiomyopathy have demonstrated incremental predictive value over clinical risk factors in large population cohorts (Inouye et al., 2018).

**TABLE 2:** Artificial intelligence and machine learning methods applied in cardiovascular genomics: categories, key algorithms, omics inputs, cardiovascular applications, and representative references.

Category	Key methods	Omics input	CVD Application	Representative references
Supervised classification	Random forest, gradient boosting (XGBoost, LightGBM), SVM	Transcriptomics, proteomics, multi-omics	Heart failure subtype classification; aortic disease risk prediction; post-operative outcome modeling	Christodoulou et al. (2019), Goldstein et al. (2017)
Polygenic risk scoring	LASSO, ridge regression, Bayesian shrinkage (LDpred2, PRSice), clumping and thresholding	GWAS summary statistics, genotyping arrays	CAD, aortic aneurysm, and cardiomyopathy risk stratification; multi-ancestry PRS	Inouye et al. (2018), Khera et al. (2018)
Unsupervised clustering	k-means, hierarchical clustering, Gaussian mixture models, consensus clustering	Transcriptomics, multi-omics panels	HFpEF vs. HFrEF molecular endotyping; atherosclerosis plaque subtyping; TAA patient stratification	Shah et al. (2015), Ahmad et al. (2018)
Multi-omics factorization	MOFA+, iClusterPlus, SNF, DIABLO, MCIA	Transcriptomics + proteomics + metabolomics + methylation	Integrated cardiovascular disease subtyping; biomarker panel discovery across molecular layers	Argelaguet et al. (2020), Shen et al. (2012)
Dimensionality reduction	PCA, t-SNE, UMAP, scVI, Harmony	scRNA-seq, spatial transcriptomics, bulk omics	Single-cell atlas construction; batch correction in multi-site CVD studies; trajectory inference	McInnes et al. (2018), Lopez et al. (2018)
Graph neural networks	GraphSAGE, graph attention Networks, graph convolutional Networks	PPI networks, co-expression graphs, molecular graphs	Drug-target interaction prediction; cardiovascular network-based drug repurposing; variant effect modeling	Zitnik et al. (2018), Huang et al. (2020)
Deep learning (images + omics)	CNNs, ResNet, vision transformers, multimodal transformers	Cardiac imaging + genomics; ECG + transcriptomics	Echo/CMR phenotyping integrated with genomics; ECG-based genetic trait discovery	Attia et al. (2019), Pirruccello et al. (2020)
Natural language/foundation models	LLMs, genomic foundation models (Nucleotide transformer, enformer)	DNA sequences, clinical notes, omics embeddings	Regulatory element prediction in CVD loci; variant pathogenicity scoring; clinical note mining	Avsec et al. (2021), Dalla-Torre et al. (2025)
Survival and time-to-event models	Cox + elastic net, DeepSurv, DRSA	Multi-omics + clinical covariates	Long-term cardiovascular event prediction; post-surgical survival modeling; heart failure progression	Katzman et al. (2018)
Causal inference/MR integration	Two-sample MR, SMR, TWAS, coloc, multivariable MR	GWAS summary statistics + eQTL/pQTL data	Causal target validation for CVD drug development; distinguishing driver from passenger molecular signals	Burgess et al. (2015), Zhu et al. (2016)

Abbreviations: CAD, coronary artery disease; CNN, convolutional neural network; DIABLO, data integration analysis for biomarker discovery using latent components; DRSA, deep recurrent survival analysis; eQTL, expression quantitative trait locus; GNN, graph neural network; GWAS, genome-wide association study; HFpEF, heart failure with preserved ejection fraction; HFrEF, heart failure with reduced ejection fraction; LASSO, least absolute shrinkage and selection operator; LDpred2, linkage disequilibrium-aware polygenic risk score method; LLM, large language model; MCIA, multiple co-inertia analysis; MOFA+, multi-omics factor analysis version 2; MR, mendelian randomization; PCA, principal component analysis; PPI, protein-protein interaction; pQTL, protein quantitative trait locus; PRS, polygenic risk score; RF, random forest; scRNA-seq, single-cell RNA sequencing; SHAP, shapley additive explanations; SMR, summary-data-based mendelian randomization; SNF, similarity network fusion; SVM, support vector machine; TAA, thoracic aortic aneurysm; TWAS, transcriptome-wide association study.

### 8.2 Unsupervised learning and molecular subtyping

Unsupervised learning approaches enable data-driven discovery of molecular subtypes within cardiovascular disease populations without requiring predefined labels. Consensus clustering of transcriptomic or multi-omics profiles has identified reproducible molecular endotypes of HFpEF, HFrEF, and aortic valve disease that correspond to distinct biological programs, clinical trajectories, and potential therapeutic vulnerabilities (Epelde, 2025). Multi-omics factorization methods, including MOFA+, iClusterPlus, similarity network fusion (SNF), DIABLO, and multiple co-inertia analysis (MCIA), enable simultaneous decomposition of variance across multiple omics layers, identifying latent factors that capture shared molecular programs underlying cardiovascular phenotypes (Argelaguet et al., 2020). These latent factors often correspond to biologically interpretable axes such as inflammatory burden, fibrotic remodeling, or metabolic dysregulation.

### 8.3 Deep learning and representation learning

Deep learning architectures have expanded the scope of AI applications in cardiovascular genomics beyond tabular omics data. Convolutional neural networks applied to cardiac imaging integrated with genomic data have enabled joint imaging-genomic phenotyping, identifying genetic determinants of cardiac structure and function at population scale (Pirruccello et al., 2020). Graph neural networks (GNNs) operate directly on network-structured omics data, enabling drug-target interaction prediction, variant effect modeling within molecular graphs, and cardiovascular drug repurposing from PPI and co-expression network embeddings (Zitnik et al., 2018). Transformer-based foundation models for genomic sequences, including Enformer and the Nucleotide Transformer, predict the regulatory activity of genomic elements from DNA sequence alone, enabling variant pathogenicity scoring at cardiovascular disease-associated loci and *in silico* characterization of non-coding regulatory variants (Avsec et al., 2021). Autoencoder and variational autoencoder architectures have been applied for omics dimensionality reduction and latent space representation of cardiovascular disease states, facilitating integration across heterogeneous data modalities.

### 8.4 Interpretability, validation, and clinical translation of AI models

A central limitation of AI applications in cardiovascular genomics is the tension between model complexity and interpretability. SHAP (SHapley Additive exPlanations) values and permutation-based feature importance methods provide *post hoc* interpretability for black-box models, enabling identification of the omics features driving predictions in clinical cardiovascular contexts (Lundberg and Lee, 2017). Beyond interpretability, the practical application of AI in cardiovascular genomics is frequently constrained by data sparsity, limited sample sizes relative to the dimensionality of omics feature spaces, and non-random missingness across assays and clinical variables, all of which can degrade model performance in ways that are not always apparent from internal cross-validation. These constraints are further complicated by the growing use of large language models (LLMs) for tasks such as literature synthesis, variant interpretation, and clinical note extraction: SHAP and related feature-attribution methods were developed for models with well-defined, fixed input features and offer substantially reduced utility when applied to LLMs, whose outputs depend on high-dimensional, context-dependent token representations that do not map cleanly onto interpretable omics features. LLMs additionally introduce failure modes distinct from conventional black-box prediction models, producing fluent but unsupported outputs, sometimes termed hallucinations, that can arise either from misreading or misrepresenting the input data provided to the model or from flawed downstream inference and reasoning over otherwise correctly parsed information; both patterns pose particular risk in genomics and clinical contexts, where fabricated gene-disease associations, citations, or variant interpretations may be difficult to distinguish from valid ones without independent verification (Anh-Hoang et al., 2025). Rigorous external validation across independent cohorts, diverse ancestries, and distinct clinical settings is a prerequisite for clinical translation, and the current literature contains numerous examples of AI models that perform well in discovery datasets but fail to replicate. Regulatory frameworks for AI-derived cardiovascular biomarkers and decision support tools remain in active development by the FDA and EMA, creating uncertainty around the clinical adoption pathway. Bridging the gap between computational prediction and clinical implementation requires not only statistical validation but functional confirmation of computationally identified targets, an area where CRISPR perturbation screens with omics readouts are emerging as a key validation tool.

## 9 Integrative multi-omics: analytical frameworks and methodological considerations

### 9.1 Vertical and horizontal integration strategies

Multi-omics integration strategies can be classified along two axes: the directionality of integration (vertical vs. horizontal) and the stage of integration (early, intermediate, or late). Horizontal integration combines data of the same omics type across different samples, cohorts, or studies, the approach underlying most large-scale transcriptomic meta-analyses and GWAS consortia. Vertical integration combines multiple distinct omics layers from the same samples or subjects, enabling identification of cross-layer molecular relationships. Early integration concatenates all omics features prior to analysis, which maximizes data utilization but is sensitive to differences in scale, sparsity, and noise across modalities (Subramanian et al., 2020). Late integration trains separate models per omics layer and combines predictions, offering robustness but sacrificing cross-modal feature interactions. Intermediate integration methods, including MOFA+, iClusterPlus, and SNF, operate at the level of learned representations or similarity networks, balancing statistical power with cross-modal interpretability. For cardiovascular applications, the choice of integration strategy should be guided by the biological question, available sample size, data completeness, and intended clinical use. In practice, this translates into a small number of recurring decision points. When the analytic cohort is small relative to the combined dimensionality of the omics layers, typically fewer than several hundred samples against thousands of concatenated features, early integration is prone to overfitting and late or intermediate integration is generally preferred, since each omics layer can be modeled with a more favorable events-per-variable ratio before combination. When missingness is substantial and unevenly distributed across omics layers, so that only a subset of samples have all modalities profiled, early integration is largely precluded because it requires a complete case matrix, whereas late integration can accommodate layer-specific missingness by training each sub-model on its own available samples and combining only the predictions. When the analytic goal is discovery of cross-layer regulatory relationships, such as identifying which methylation or chromatin changes drive a co-expression module, intermediate integration methods that preserve a shared latent representation are preferable to late integration, which discards cross-modal interactions by design. Finally, when the intended output is a clinical decision-support tool, robustness to a missing or delayed modality at the point of care, for example a patient without same-day proteomic data, favors late integration architectures that degrade gracefully rather than early integration architectures that require a fixed, complete input vector.

### 9.2 Causal inference: mendelian randomization and beyond

A critical challenge in integrative genomics is distinguishing causal molecular drivers from downstream biomarkers or confounded associations. Mendelian randomization (MR) uses genetic variants as instrumental variables to infer causal relationships between molecular phenotypes, such as gene expression, protein abundance, or metabolite levels, and cardiovascular outcomes (Yazdani et al., 2022). Two-sample MR leverages large GWAS and molecular QTL summary statistics from non-overlapping populations, enabling causal inference at genome-wide scale without individual-level data (Burgess et al., 2015). Summary-data-based MR (SMR) and transcriptome-wide association studies (TWAS) extend causal inference to gene expression, while colocalization analysis (coloc) tests whether a single causal variant underlies both a molecular QTL signal and a GWAS disease association, distinguishing causal from LD-confounded relationships (Zhu et al., 2016). Multivariable MR further enables simultaneous estimation of the direct causal effects of multiple correlated molecular exposures, an approach particularly relevant to the highly interconnected molecular landscape of cardiovascular disease. These causal inference frameworks, when integrated with network biology approaches, provide the highest-confidence strategy for identifying therapeutically actionable cardiovascular targets supported by human genetic evidence.

### 9.3 Challenges: batch effects, confounding, and data harmonization

The practical implementation of multi-omics integration in cardiovascular research faces substantial methodological challenges. Batch effects, systematic technical variation introduced by differences in sample processing, sequencing platform, reagent lot, or laboratory, can dominate biological signal in multi-site omics studies and must be carefully addressed prior to integration (Leek et al., 2010). Computational batch correction methods including ComBat, Harmony (for single-cell data), and scVI have been widely adopted, but none eliminates the need for careful study design and metadata documentation. Biological confounders, including age, sex, ancestry, medication use, and comorbidity burden, require rigorous adjustment in cardiovascular multi-omics analyses, particularly when integrating datasets from heterogeneous clinical populations. Missing data is a pervasive challenge in multi-omics cardiovascular studies, as different assays are rarely performed on exactly the same samples; imputation methods and analysis frameworks designed for incomplete multi-omics data are an area of active methodological development (Meng et al., 2016). Adherence to FAIR data principles (Findable, Accessible, Interoperable, Reusable) and adoption of community-standard data formats are essential for enabling the cross-study integration that maximizes the value of cardiovascular omics investments. Several additional methodological barriers are particularly relevant to cardiovascular multi-omics research. Tissue and cell-type specificity, combined with the limited availability of disease-relevant human cardiovascular tissue, constrains the generalizability of findings derived from surrogate tissues or animal models. Temporal variation across disease stages and treatment exposure means that a single cross-sectional omics snapshot may not represent the trajectory of aneurysm growth, cardiac remodeling, or valve degeneration, while replication and external validation across independent cohorts remain inconsistently performed. Ancestry and population diversity, clinical phenotype heterogeneity, and confounding by medication use and comorbidity burden can each masquerade as biological signal if not explicitly modeled, and even well-harmonized datasets face the more fundamental difficulty of distinguishing causal molecular mechanisms from signatures that are merely correlated with disease state. A related and often underappreciated barrier lies upstream of integration itself, in data preparation. Raw omics data typically require cleaning, normalization, and standardization, for example variance-stabilizing transformation of RNA-seq counts, batch-aware normalization of mass spectrometry proteomics, and quality-control filtering of single-cell data for doublets and ambient RNA, before any network or AI-based model is applied; deficiencies at this preprocessing stage propagate through every downstream analysis and can produce spurious network structure or model performance that fails to generalize. Achieving adequate statistical power and representativeness for these preprocessing and integration steps increasingly requires pooling data across multiple institutions, which in turn raises patient privacy and data-governance constraints that limit centralized data sharing. Federated learning, in which a shared model is trained iteratively across institutional datasets while patient-level data remain local, addresses this tension directly and is discussed further, together with its application to network biology, in Section 7.6 (Rieke et al., 2020).

## 10 Translational applications and precision cardiovascular medicine

### 10.1 Biomarker discovery and validation

Integrative multi-omics approaches have substantially expanded the cardiovascular biomarker discovery landscape. Multi-omics-derived biomarkers, such as the apolipoprotein-B:A1 ratio identified in cardiometabolic risk profiling of juvenile-onset systemic lupus erythematosus (Robinson et al., 2021), demonstrate the clinical utility of integrative molecular profiling beyond standard single-analyte assays. Multi-omics analyses of extracellular vesicles further demonstrate how circulating biomolecules can reflect disease-associated transcriptional and proteomic network alterations, supporting non-invasive biomarker discovery and disease stratification in cardiovascular pathology (Meng et al., 2024). Protein biomarker panels derived from large-scale affinity proteomics, including panels predictive of incident heart failure, atrial fibrillation, and cardiovascular mortality in the UK Biobank Pharma Proteomics Project, represent an emerging paradigm for precision cardiovascular risk assessment (Sun et al., 2023). Rigorous biomarker validation requires demonstration of analytical validity, clinical validity across diverse populations, and ideally clinical utility in prospective intervention studies, a pathway that most multi-omics-derived biomarkers have yet to complete.

### 10.2 Drug target prioritization and repurposing

Integrative genomics has fundamentally restructured the cardiovascular drug discovery pipeline by enabling hypothesis-free, evidence-based target prioritization. Targets supported by human genetic evidence, particularly those with MR or pQTL evidence for causal effects on cardiovascular outcomes, have substantially higher clinical success rates than targets identified through preclinical biology alone (Nelson et al., 2015). Network proximity-based drug repurposing frameworks systematically identify approved drugs whose targets lie in close topological proximity to cardiovascular disease modules, flagging repurposing opportunities with mechanistic plausibility (Guney et al., 2016). Examples of genomics-informed cardiovascular drug successes include the development of PCSK9 inhibitors, guided by loss-of-function variant evidence from GWAS (Cohen et al., 2006), and the emerging development of ANGPTL3 inhibitors supported by population genetics (Dewey et al., 2017). The integration of transcriptomic, proteomic, and network evidence with genetic causal inference creates a multi-evidence scoring framework that can prioritize the highest-confidence targets from the long lists generated by omics discovery analyses.

### 10.3 Polygenic risk stratification and precision prevention

Polygenic risk scores (PRS) aggregate the effects of thousands to millions of genetic variants across the genome to provide an individual-level estimate of genetic disease liability. PRS for coronary artery disease, now derived from GWAS including over one million participants, demonstrate incremental predictive value over traditional clinical risk factors and have been validated in multiple independent cohorts (Inouye et al., 2018; Patel et al., 2023). PRS for thoracic aortic aneurysm, aortic valve disease, and cardiomyopathy are in active development, and their integration with imaging and biomarker data is expected to refine risk stratification for these conditions. A major priority is the extension of PRS to non-European ancestries: current PRS models trained predominantly on European ancestry GWAS underperform in individuals of African, Asian, and admixed ancestry, limiting their equitable clinical application (Martin et al., 2019). Multi-ancestry GWAS and ancestry-specific LD reference panels are being developed to address this gap, and trans-ethnic PRS methods are emerging as a priority area for the field. The All of Us Research Program (Section 3.1) is particularly well positioned to advance this effort: because it links whole-genome sequencing to electronic health records, survey data, and physical measurements across a cohort in which the majority of participants come from communities historically under-represented in genomics, it enables direct empirical evaluation of PRS transferability rather than reliance on European-derived scores alone. Analyses combining All of Us with the UK Biobank have shown that increasing cohort diversity improves PRS accuracy specifically for under-represented individuals, and that for some traits with large ancestry-enriched genetic effects, training on the more diverse All of Us data alone outperforms simply maximizing sample size across biobanks, underscoring that representativeness, not sample size alone, determines equitable PRS performance (Tsuo et al., 2024).

### 10.4 Single-cell resolution and spatially resolved Precision Medicine

The emergence of single-cell and spatial omics technologies has created new opportunities for cell-type-specific therapeutic targeting in cardiovascular disease. By identifying disease-associated cell states, such as the osteogenic VIC phenotype in calcific aortic valve disease or the GPNMB-high macrophage in TAA, single-cell analyses define precise molecular targets expressed selectively within pathological cell populations, minimizing off-target effects on healthy cardiovascular tissues (Qin and Bäck, 2026; Zhao et al., 2025). Spatially resolved transcriptomics further identifies the anatomical context of disease-associated gene programs, enabling the design of locally acting therapeutic strategies and informing decisions about surgical tissue sampling and *ex vivo* molecular profiling. The application of these technologies to surgical biobanks, including aortic, valvular, and myocardial specimens obtained during planned cardiac operations, represents an immediate and practically accessible opportunity to generate mechanistically rich datasets with direct clinical context.

## 11 Future directions

The integration of omics technologies with network biology and artificial intelligence is rapidly transforming cardiovascular research from descriptive molecular profiling toward mechanistic, predictive, and ultimately prescriptive frameworks. Several priority directions emerge from this synthesis.

First, the expansion of cardiovascular single-nucleus RNA-seq and spatial transcriptomics from surgical specimens, including aortic, valvular, and myocardial tissues obtained at the time of planned operations, represents an immediate opportunity to generate mechanistically rich datasets with direct clinical context, linking intraoperative molecular profiles to longitudinal outcome data. Second, federated learning architectures offer a pathway to leverage distributed multi-institutional omics data without centralizing sensitive patient information, addressing both privacy and data harmonization challenges while enabling the large effective sample sizes required for robust multi-omics modeling (Rieke et al., 2020). Third, the development of continuously updated, computationally driven living guidelines, integrating real-time omics evidence with clinical outcome data, represents a promising frontier for translating network biology insights into dynamic clinical decision support at the point of care.

Fourth, functional genomics validation pipelines combining CRISPR perturbation screens with omics readouts will be essential for moving from statistically identified network hubs to mechanistically validated therapeutic targets, closing the gap between computational discovery and experimental confirmation (Shan and Fei, 2023). Fifth, the establishment of multi-ancestry omics reference datasets and PRS validation frameworks is critical to ensuring that the benefits of integrative genomics are equitably extended across diverse patient populations. Finally, the progressive adoption of multimodal AI architectures, integrating genomic, imaging, clinical, and wearable sensor data, is expected to unlock precision cardiovascular medicine capabilities that are currently inaccessible to single-modality approaches. Addressing these priorities will require sustained investment in computational infrastructure, interdisciplinary collaboration, and rigorous frameworks for transitioning from computational prediction to clinical implementation.

## 12 Conclusion

Integrative genomics and network biology represent a paradigm shift in cardiovascular research, moving the field from single-gene or single-pathway thinking toward a systems understanding of disease. The convergence of transcriptomics, epigenomics, proteomics, metabolomics, and network-based analytical frameworks, enabled by expanding public omics repositories and computational tools, has produced mechanistic insights into thoracic aortic aneurysm, heart failure, valvular disease, and vascular remodeling that were inaccessible through traditional approaches. Artificial intelligence is increasingly serving as the connective tissue between these modalities, enabling pattern recognition, causal inference, and predictive modeling at scales and resolutions beyond conventional statistics. Yet the translational gap between network-level discovery and clinical implementation remains substantial. Rigorous external validation, functional confirmation of computationally identified targets, and the development of multi-ancestry reference standards are prerequisites for responsible clinical translation. The systems-level framework outlined in this review, spanning omics data generation, network biology analysis, AI-driven modeling, causal inference, and translational validation, is intended to guide investigators in navigating these methodological opportunities and challenges, and to catalyze the next-generation of discovery-to-implementation research in cardiovascular genomics.
